# Rumour prevention in social networks with layer 2 blockchains

**DOI:** 10.1007/s13278-021-00819-y

**Published:** 2021-10-21

**Authors:** Subhasis Thakur, John G. Breslin

**Affiliations:** National University of Ireland, Galway, Ireland

**Keywords:** Social networks, Rumour, Blockchains, Blockchain offline channels, Social networks (91D30), Blockchains (94A60), Blockchain offline channels (68P25)

## Abstract

Social bots can cause social, political, and economical disruptions by spreading rumours. The state-of-the-art methods to prevent social bots from spreading rumours are centralised and such solutions may not be accepted by users who may not trust a centralised solution being biased. In this paper, we developed a decentralised method to prevent social bots. In this solution, the users of a social network create a secure and privacy-preserving decentralised social network and may accept social media content if it is sent by its neighbour in the decentralised social network. As users only choose their trustworthy neighbours from the social network to be part of its neighbourhood in the decentralised social network, it prevents the social bots to influence a user to accept and share a rumour. We prove that the proposed solution can significantly reduce the number of users who are share rumour.

## Introduction

Social Bots are autonomous software agents who promote or demote social media content with specific sentiments in online social networks (OSN). Social bots can be used to promote rumours in OSN. A rumour can be initiated by a social bot or a malicious user who publishes social media content with false information to cause social, political (https://www.cheq.ai/fakenews) and economical (https://seekingalpha.com/article/ 876 4129355-cost-of-fake-news-for-s-and-p-500) disruptions. Social bots can create or share a rumour and give positive feedback, comments, etc. to promote its propagation in the OSN.

The state-of-the-art methods to prevent rumour propagation include (1) identification of rumour sources, (2) automated detection of rumours, and (3) censorship of social media content and users by the OSN operator. These methods can be useful for centralised solutions. In a centralised method to prevent social bots from spreading the OSN operator may deploy a machine learning-based algorithm to detect rumour and censor users to stop its propagation. However, the OSN operator may be biased and selectively stop rumours with specific sentiments. There are several biased content vetting incidents (https://www.pewresearch.org/internet/2020/08/19/ 884 most-americans-think-social-media-sites-censor-political-885 viewpoints/, https://www.887 bbc.com/news/technology-54698186). In this paper, we propose a decentralised method that can prevent social bots from spreading rumours. We use a public proof of work-based blockchain to develop such a decentralised solution. In this solution, the users of an OSN form a decentralised social network (DSN) with their trusted neighbours from the OSN. The trusted neighbours are unlikely to promote a rumour. A user can verify if the content is shared by its neighbours in the DSN before sharing the content by itself, and it prevents the social bots from influencing a user. There are few problems with this approach are as follows:

- Public blockchain has a scalability problem. Hence it is necessary to address this problem while we use public blockchains to support content propagation in social networks.
- A malicious entity would like to know the trusted neighbours of a user, i.e. its neighbourhood in the DSN, and influence the user by controlling (possibly via malware) its trusted contacts.
- A user may not participate in the proposed mechanism and may be influenced by anyone in its social neighbourhood.

Our main contribution in this paper solves these problems as follows:

- We use blockchain offline channels to build a high-scale solution. An offline channel only creates two blockchain transactions to support a high number of transactions between two peers. It reduces the number of transactions in the blockchain to make it scalable.
- We have developed a content propagation that does not allow a malicious entity to discover the DSN neighbourhood of each user by analysing the transactions in the blockchain offline channels which is used to implement the content propagation method. The proposed offline channel-based content propagation allows a user to ‘buy’ a content and ‘sell’ it to its neighbours as it shares it. We have developed an offline channel-based privacy-preserving and secure protocol for such buy and sell operations.
- The proposed protocol allocates a finite number of tokens to each user which they use for the propagation of content. We prove that the irrational users who are influenced by anyone in its neighbourhood loses too many tokens for their continued participation in content dissemination.

Our results in this paper are as follows:

- A privacy-preserving DSN:
  We developed a method to create a DSN from an OSN using a public blockchain (Bitcoin) network. We developed a secure and privacy-preserving method to share social media content via the DSN where the social neighbourhood of a user in the DSN can be hidden. By hiding the social neighbours of a user in the DSN, the proposed solution prevents the social bots to target and compromise influential users.
- Incentives for choosing social-bot free neighbours in the DSN:
  We developed a token-based content propagation model where a user has to buy content before it can sell the social content to other neighbours. Our solution allocates a finite number of tokens to each user and if a user cannot sell content then it losses token. We show that the users who do not carefully choose their neighbours in the DSN lose more tokens. It prevents such users to share social media content as they do not have sufficient tokens.
- Analytical evaluation:
  We prove that the proposed solution is privacy-preserving as social bots will not know the neighbours of the users in the DSN and the proposed solution is secure as social bots cannot circumvent the token-based content propagation to spread rumours. We prove that the proposed solution is correct as the balance in the channels owned by irrational users (who do not differentiate between a social bot and a genuine OSN user) becomes too low to be used for content spreading.
- Experimental evaluation:
  We evaluated the performance of the proposed solution using simulations of social media content propagation. We found that the proposed solution prevents the propagation of rumours.

The paper is organised as follows: in Sect. 2 we discuss related literature, in Sect. 3 we discuss the problem of social bot facilitated rumour propagation, in Sect. 4 we describe our decentralised method to prevent social bots from spreading rumours, in Sect. 5 we present an analysis of the proposed solution, in Sect. 6 we present an experimental evaluation of the proposed solution and we conclude the paper in Sect. 7.

## Related literature

In Tong et al. (2017) propagation of correct information to counter rumour is proposed. Takayasu et al. (2015) analysed rumour creation during natural disasters. Shao et al. (2016) proposed an online platform for fact-checking to prevent rumour propagation in social media. McCreadie et al. (2015) proposed to use crowdsourcing to prevent rumours during emergencies. Liu et al. (2015) proposed a method for real-time detection of rumours. Liang et al. (2015) proposed to analyse user behaviour for rumour detection. Lendvai and Reichel (2016) proposed a novel method for rumour detection by identifying contradictions. Kwon et al. (2017) used time windows for rumour detection. Detecting rumour and the source of the rumour is well researched and several algorithms are developed (Pathak et al. 2020; Zubiaga et al. 2018) to detect rumours. Rumour detection techniques have used machine learning-based algorithms. Identifying the source of rumour Jiang et al. (2018) can also be an effective tool to deter malicious entities from creating misinformation in social networks. In Alzanin and Azmi (2018) the authors presented a survey on rumour detection methods for social networks. A similar survey was presented in Zubiaga et al. (2018). In this paper, we develop a rumour prevention mechanism using content vetting by the users.

Blockchain is recently applied to design several social media platforms. SteemIt Kiayias et al. (2018) is a blockchain-based online social media platform that rewards its users for creating and rating new content. SteemIt uses Steem (Li and Palanisamy 2019), which uses delegated proof of work (https:// 879steemit.com/dpos/@dantheman/dpos-consensus-algorithm-880 this-missing-white-paper) and is more scalable than a proof of work-based blockchains. Lit (https://mith.io/en-US/) is a blockchain-based social network platform that is developed using Ethereum. Users are rewarded for creating content in this social media platform, and the amount of reward depends on the popularity of the content. SocialX (https://socialx.network/wp-content/uploads/2018/09/ Whitepaper-SocialX-v1.1.pdf) is a decentralised social media platform designed to deter fake users from a social media platform. SocialX uses Ethereum as the blockchain. Users are rewarded for checking the validity of media content. Foresting (Foresting: Rewarding lifestyle social media 2019) is a blockchain-based social media platform where users are rewarded for creating valuable content, and the usefulness of content is judged by users of the Foresting network. Minds (Minds: The crypto social network 2019) is an Ethereum-based social media platform that guarantees that there is no censorship of the content created in this social media platform. Decentralisation of the social media platform immunes content from censorship. Minds platform uses both on-chain or off-chain transactions. Guidi (2020) presented a detailed characterisation of these social media platforms. Jiang and Zhang (2019) developed a blockchain-based DSN. In this social network, user data are kept in the blockchain and a user can modify and delete its data. Additionally, this DSN uses attribute-based encryption to preserve the privacy of the users, and as such encryption allows access to only a subset of the user data. In Xu et al. (2018) a Blockchain and IPFS based DSN model was proposed. It uses Ethereum smart contracts to develop DSN functionalities. Ur Rahman et al. (2020) developed a blockchain-based DSN with Ethereum as the blockchain. It uses Ethereum smart contracts for access control over the user data in this DSN. Bahri et al. (2018) analyses the security and privacy challenges in developing a DSN platform. Dongqi and Fang (2016) used blockchains for privacy-preserving data management in social networks. Yang et al. (2020) provided a survey on blockchain-based social networks and social media. Guidi et al. (2020) analyse reward models for users in DSN. Freni et al. (2020) discussed how blockchain can solve privacy and security problems with OSN. Yang et al. (2020) proposed a blockchain-based secure friend matching algorithm for OSN.

In Comito (2020), an algorithm to predict the user’s location in location-based social networks is presented. In Comito et al. (2019) an algorithm to identify social neighbourhood using word embedding is proposed. Salem et al. (2011) proposed a community discovery algorithm using network topology. Schreckenberger et al. (2018) presents a survey on predicting the next location of users in location-based social networks. Chunaev (2020) presents a survey on community detection in social networks. The research on community detection or next location prediction is a threat to the decentralised social network (where the users want to hide their social neighbourhood) proposed in this paper. However, the proposed DSN formation is secure against these algorithms as transactions in the blockchain offline channel network will not reveal the social neighbourhood structure of the DSN. In this paper, we use proof of work-based blockchains. It was proposed in Nakamoto (2008). There are several variations of blockchains in terms of consensus protocols. Bitcoin lightning network was proposed in Poon et al. (2016) which allows peers to create and transfer funds among them without frequently updating the blockchain.

Our contributions advance the state-of-the-art in securing social networks in the following directions:

- Most of the existing solutions for rumour prevention are focused on rumour detection problems. These solutions model rumour with various features and use machine learning to predict if social media content is a rumour. But, in this paper, we allow the users of the social network to decide if the content is a rumour and developed incentives for the users to correctly classify the social media content.
- We have developed a decentralised method to prevent social bots from spreading rumours. In the existing rumour prevention methods, rumour is first detected and then a centralised authority (the OSN operator) prevents propagation of the identified rumour. This approach has significant problems as the OSN operator from selectively censoring rumours. The proposed decentralised content vetting solution may prevent such biased rumour prevention.
- The proposed solution is secure and privacy-preserving as a user will not the another user’s neighbourhood in the DSN. The existing rumour prevention solutions rarely investigate the privacy and security problems of rumour prevention algorithms.
- There are several algorithms to detect a user’s community. This can be used to identify the DSN neighbourhood. But we prove that it is not possible to identify DSN user’s social neighbourhood by analysing the offline channel transactions.

## False information propagation with social-bots

### Content propagation model

We will use a cascading model of information propagation (Guille et al. 2013) in social networks. In this model of content propagation, initially, one user of the social network creates the content and shares it with its neighbours. Next, these neighbours may share it with their respective neighbours. This process continues until either all users receive the content or all users who had received the content has decided if they want to share it with their neighbours. In the cascading model of content propagation, there are two types of network topology of social networks:

1. Restricted Cascading Network: In this network, a user *A* will choose its incoming and outgoing edges in the social network, i.e. it selects the users from whom it will receive content via the cascading procedure and it also selects the users to whom it will share content. This social network is similar to the Facebook social network.
2. Unrestricted Cascading Network: In this network, a user *A* will only choose its incoming i.e. it selects the users from whom it will receive content via the cascading procedure. But any other user can receive content from *A* by creating an outgoing edge from *A* to itself in the social network. This social network is similar to the Twitter network where anyone can follow anyone, i.e. a user does not restrict the creation of outgoing edges from it.

Further, we will use the influence maximisation model (Kempe et al. 2003) of content propagation. In this model, a user $v_i$ will share content with its neighbours if it has received the content from at least $\tau$ fraction of its neighbours. $\tau \in [0,1]$ is chosen by the user (This value represents the likelihood that a user can be influenced by its neighbours.). $\tau$ shows the difficulty to influence a user. We will use the following model of content propagation:

1. If a user receives content from at least $\tau$ fraction of neighbours then, it will share the content with its neighbours.
2. Otherwise, it will not share the content.

**Fig. 1:**
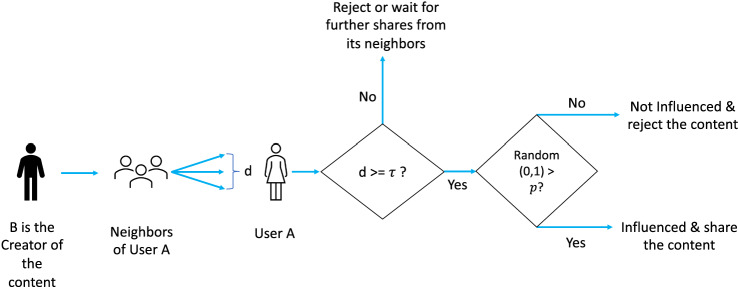
Content propagation model used in this paper uses cascading and influence maximisation

A weighted version of the content propagation model with influence maximisation is as follows:

1. Each user assigns a weight between 0 and 1 to all of its neighbours in the OSN such that for all users, the sum of all such weight is assigned to its neighbours 1.
2. A user will calculate $\tau$ as weighted fraction of its neighbours, i.e. $\sum _x W(x,v) / n$ where *x* is a neighbour of the user *v* who has shared the content and *W*(*x*, *v*) is the weight of the edge from *x* to *v*.
3. If a user receives content from at least $\tau$ fraction of neighbours then it will share the content with its neighbours.
4. Otherwise it will not share the content (Fig. 1).

### Content propagation with social-bots

Social-bots can be used by the adversarial user to propagate misinformation and prevent the propagation of correct information. Social-bots can be part of the social neighbourhood of genuine users. Additionally, social-bots may use malware to control information to and from a genuine user. A social bot will share a rumour irrespective of the value of $\tau$. Social bots are assumed to be controlled by an adversarial entity and they can identify content as a rumour it wants to spread.

## Preventing social bots with blockchains

### System model and assumptions

The OSN is represented as a directed graph $G = (V,E)$ with a set of vertices $V = (v_1,\dots ,v_n)$ representing the set of users of the OSN, and the set of edges represent the social neighbourhood of the OSN users. Let *BC* be a blockchain network with set of peers $P = (p_1,\dots , p_x)\cup (O_1,\dots ,O_k)$. Let *BC* is a public blockchain and it uses proof of work as the consensus model. The OSN operator operates *k* accounts $O = (O_1,\dots ,O_k)$ in this blockchain. The OSN operator publishes these *k* blockchain accounts by maintaining a secure list of these accounts (with their public keys). This means an adversary cannot create a blockchain account and claim it is part of the set *O*. We assume that these accounts cannot be controlled by an adversary. All users of the OSN may operate a unique account in the blockchain network. $p_i$ will represent the blockchain account of the OSN user $v_i$. An OSN user may have multiple accounts in the blockchain by the OSN will recognise only one such account per one user. The OSN will maintain a secure list of such blockchain accounts (public keys) of the users. We assume that an adversary cannot alter such a list but it may control such blockchain account of the OSN users. The adversary may use malware to reveal the private key of a user corresponding to its registered blockchain account (public key) to the OSN.

**Fig. 2:**
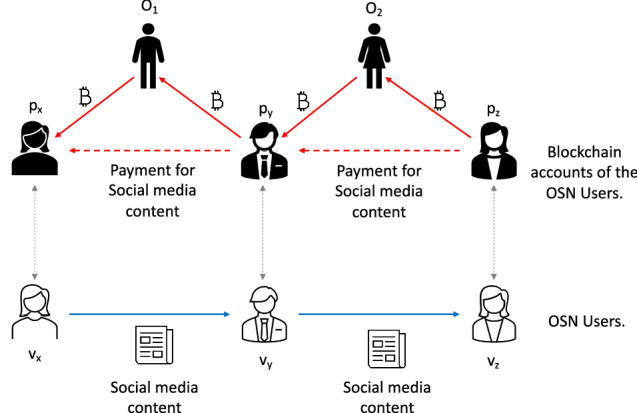
Overview of the proposed rumour prevention solution. A user ‘buys’ content and sells it to others as it shares the content. It is assumed that rational users will not ‘buy’ rumours, and the social bots or irrational users who ‘buys’ a rumour will not be able to sell it. We used offline channels to implement this content trading process

The OSN operator will establish a permissioned blockchain offline channel network from the public blockchain *BC *(explained in Fig. 2). The offline channel network consists of the blockchain accounts *O* and registered blockchain accounts of the OSN users. The network is permissioned as the OSN operator will verify that an OSN user operates and controls a blockchain account. We will use unidirectional channels (explained in the next section) to establish these offline channels. There will be two types of channels in this offline channel network. One, between an OSN user and the OSN operator, and another between two OSN operators. Operating an offline channel requires at least one multi-signature address between two parties of an offline channel. We need to create a transaction in the blockchain with a positive amount of tokens in the blockchain network to open an offline channel. In these offline channels, there will be two types of channels between a user $v_i$ and an OSN operator $O_j$. One channel from $v_i$ to $O_j$ (which allows $v_i$ to pay $O_j$) and another channel from $O_j$ to $v_i$ (which allows $O_j$ to pay $v_i$). We will assume that the OSN operator uniformly funds all channels with a fixed amount, i.e. the OSN operator transfers funds to the multi-signature addresses between $v_i$ and $O_j$ for both channels (to and from $O_j$).

This offline channel network will be used to authenticate that a user may share content only if it has received it from its trusted contacts. The trusted contacts of each user will be represented as the DSN. In the DSN, each user will choose its trusted contacts in the OSN to be part of its social neighbourhood in the DSN. The DSN will be privacy-preserving as only a user will know its social neighbours in the DSN. A user will be encouraged to share content only if a sufficient number of its neighbours in the DSN have shared it. We will assume that rational users of the OSN will not share a rumour and each user should carefully choose its neighbours in the DSN to exclude the social bots. Thus rumour propagation will be decreased if each user correctly chooses its neighbours in the DSN. We will implement a procedure that will restrict the OSN users from sharing content that does not exclude the social bots from its neighbourhood in the DSN.

We will implement the following procedure to share content. A user will ‘buy’ content which it can ‘sell’ to others. We will use the offline channels to implement such a trade. A user can verify that seller (another user) has ‘bought’ the content before buying content. We will use ‘proof of path’ (explained in the next section) for such verification. ‘Proof of path’ verifies that a user bought content before selling it to others. Thus if a user incorrectly chooses social bots as its neighbours in the DSN and buys a rumour then it may not sell it to rational users. This will gradually decrease the channel balance of the user and eventually, it will not be able to buy content.

We have the following adversaries:

- Social bots: Who promote rumours and can coordinate their actions to promote rumours. Social bots may use malware to control OSN users. Such affected OSN users will be considered as part of the social bots. Social bots can also attempt to transfer funds to each other’s channel to propagate content. Social bots want to reveal the DSN neighbourhood to control trusted neighbours of users to influence it.
- Irrational users: Who include social bots in their DSN neighbourhood, and they will share any content even if it is a rumour.

In presence of these adversaries we have the following design goals: In the above-described procedure to prevent rumour propagation by social bots, we need to ensure the following:

- Guaranteed expiry of channels:
  As payments are made via offline channels we need to ensure that an offline channel only allows a fixed finite number of transfers. General offline channels such as the Lightning network of Bitcoin allow a potentially infinite number of transfers.
- Privacy of DSN:
  We need to ensure that, a social neighbourhood of a user in the DSN remains a private information. If a social bot can identify a user’s trusted neighbours in the DSN then it may gain control of such neighbours (via malware) to influence the user.
- Privacy-preserving verification of content purchase:
  We need to ensure that a user can verify that a seller has bought the content in a privacy-preserving fashion, i.e. without knowing who sold the content to the seller.
- Security of proof of content purchase:
  We need to ensure that a seller’s claim that it has bought the content from another user is secure, i.e. it can not lie about it or there must be proof that it had paid for it.
- Security of channels:
  We need to ensure that social bots can not transfer funds among themselves to support the propagation of a rumour. This means we need to ensure that transfers in the offline channel network are supervised by the OSN operator and a user can not pay another user without involving at least one OSN operator.
- Correctness of the solution:
  We need to prove that the proposed content share model decreases the channel balance of the users who have social bots in their DSN neighbourhood.

In this section, first, we will explain uni-directional blockchain offline channels. Next, we will explain how the users can form the DSN using the uni-directional offline channel. Then, we will describe the protocol for content propagation via the DSN which can prevent the social bots from spreading rumours.

### Unidirectional offline channel

Blockchain offline channels (Poon et al. 2016) uses multi-signature addresses to open an offline channel among peers of the blockchain. This offline channel (Poon et al. 2016) is bidirectional and potentially infinite, i.e. it can execute the infinite number of transfers between two peers provided they do not close the channel and each of them has sufficient funds. We construct an offline channel for proof of work-based public blockchain with the following properties:

- We construct a uni-directional channel between two peers, i.e. only one peer can send funds to another peer of this channel.
- We construct a uni-directional channel that can be used for a finite number of transfers from a designated peer to another peer.

**Fig. 3:**
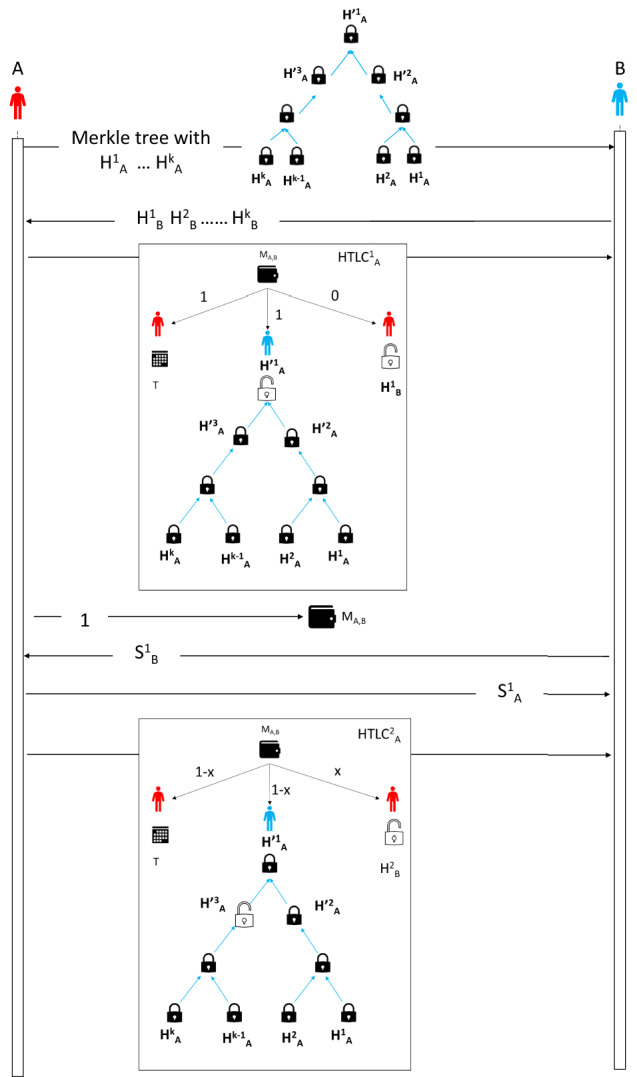
Protocol to create uni-directional offline channels

The procedure for creating the uni-directional channel (shown in Fig. 3) from *A* to *B* (*A* transfers token to *B*)is as follows: Let *A* and *B* are two peers of the channel network *H*. $M_{A,B}$ is a multi-signature address between *A* and *B*. This is a unidirectional channel from *A* to *B*.

1. *A* creates a set of *k* (*k* is a positive even integer) random strings $S_A^1,\dots ,S_A^{k}$. Using these random strings *A* creates a set of Hashes $H^1_A = H(S_A^1), H^2_A = H(S_A^2)\dots , H^{k}_A = H(S_A^{k})$ where *H* is Hash function (using SHA256). *A* creates a Merkle tree order $\lambda$ using these Hashes. Thus there are *k* leaf nodes and $k-1$ non-leaf nodes of this Merkle tree. We denote the non-leaf nodes as $H'^1_A,\dots , H'^{(k-1)}_A$.
2. *B* creates a set of *k*1 random strings $S^1,\dots ,S^{k}$ and corresponding Hashes $H^1_B,\dots ,H^k_B$.
3. *A* sends the Merkle tree to *B* and *B* sends the set of Hashes $H^1_B,\dots ,H^k_B$ to *A*.
4. *A* sends a Hashed time-locked contract $HTLC^1_A$ to *B* as follows:
  - From the multi-signature address $M_{A,B}$, 1 token will be given to *A* after time *T* if *B* does not claim these tokens before time *T* by producing the key to $H'^1_A$ and 0 token will be given to *A* if it can produce the key to $H^1_B$.
  - *A* sends $$HTLC^1_A$$ to *B*.
5. Now, *A* sends 1 token to $M_{A,B}$. *A* includes the Merkle tree and $H^1_B,\dots ,H^k_B$ in this transaction. This records the Merkle tree and $H^1_B,\dots ,H^k_B$ in the blockchain and any other peer can verify the existence of these Hashes by checking transactions of the public blockchain. Also, at this stage, *A*’s funds are safe as it can get the tokens from $M_{A,B}$ after time *T* as *B* does not know $H'^1_A$.
6. Next to send another (1/*k*) tokens to *B*, *A* sends $S^1_A$ to *B* and *B* sends $H^1_B$ to *A*. Then *A* forms the following HTLC:
  - From the multi-signature address $M_{A,B}$, $1-1/k$ token will be given to *A* after time *T* if *B* does not claim these tokens before time *T* by producing the key to $H'^2_A$ and 1/*k* token will be given to *A* if it can produce the key to $H^2_B$.
  - *A* sends $$HTLC^2_A$$ to *B*.
7. This process continues until all keys of the Hashes of non-leaf nodes are revealed by *A*.

In this model of the unidirectional channel, *A* is sequentially releasing the keys of the Merkel tree of the HTLCs. Its fund in this channel is decreasing with time. It can not prevent *B* from obtaining the tokens as only *B* can publish the HTLCs. *B* will publish the HTLC where it gets the maximum value.

### DSN creation with uni-directional channels

A DSN is a social network with the following properties:

1. Social network operations, such as content forwarding, searching for contacts, etc. are executed in a decentralised platform.
2. Each user only knows its social neighbourhood. A user can not know other user’s social neighbourhood even if they are neighbours to each other.
3. A secure content propagation model through a DSN allows a user to verify that the content followed a path from the creator of the content to itself without knowing the neighbourhood of the users.

**Fig. 4:**
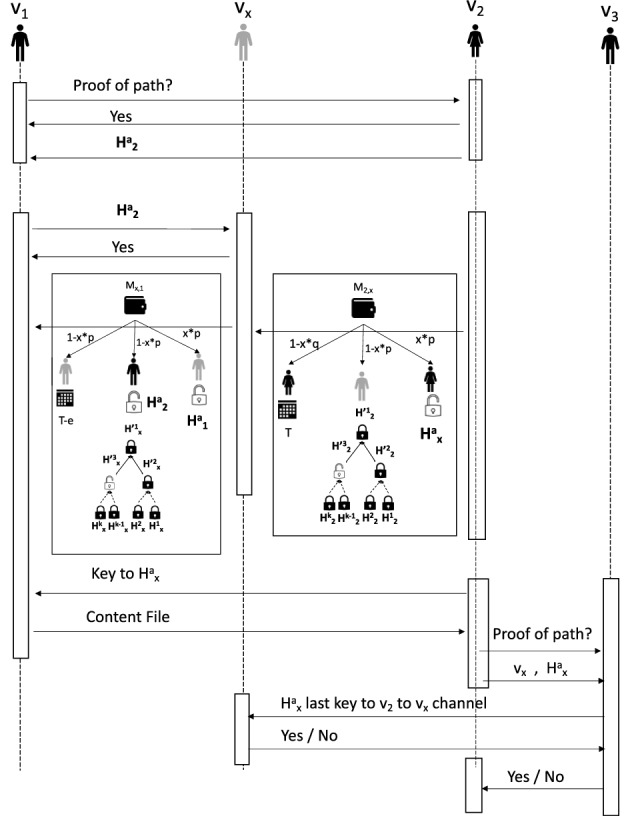
Procedure to create DSN and content propagation

The DSN formation protocol and content propagation via the DSN are as follows (explained in Fig. 4):

1. Any user of the OSN should be part of a blockchain network if the user wants to be part of the DSN. We will assume Bitcoin is the blockchain network. The OSN operator will also participate in the blockchain network with several accounts, i.e. it will be represented by several nodes of the blockchain network.
2. Any user who wants to be part of the DSN will establish a unidirectional channel with a subset of blockchain peers controlled by the OSN operator. Nodes controlled by the OSN operator may establish a unidirectional channel among themselves.
3. The DSN will consist of the above-mentioned unidirectional channels in the blockchain network.
4. A user $v_1$ of the OSN will choose its trusted neighbour $v_2$ in the OSN to be part of the DSN if both $v_1$ and $v_2$ are part of the blockchain network. $v_2$ can prove that it has received content from one of its neighbour in OSN who is also its neighbour in the DSN by proving that there was a transaction in the blockchain unidirectional network which started from $v_2$ and ended at $v_1$ via the nodes controlled by the OSN operators.

### Channel network creation

Initially, each user of the OSN will be allowed to establish a fixed and finite number of unidirectional offline channels with the OSN operator nodes in the blockchain network. The characteristics of such offline channels are as follows:

1. Every user establishes a fixed number of channels ($n_o$) with the OSN operator nodes in the blockchain. These channels start from an OSN user and end to an OSN operator.
2. Every user establishes a fixed number of channels ($n_i$) with the OSN operator nodes in the blockchain. These channels start from an OSN operator and ends to an OSN user.
3. Each channel has *z*/*k* tokens and the amount of each transfer is 1/*k* tokens. Hence each channel can be used for $z*k$ transfers.
4. The OSN operator makes the initial payment to open all channels. The OSN operator nodes will establish an offline channel network among themselves.
5. The network topology of this offline channel network is known to all users of the blockchain. It is assumed that this network does not support other payment services for the blockchain, i.e. it cannot be used for payment for other services.

### Content propagation with DSN

In this proposed content propagation model, a user will buy content before it shares the content with others. The users will use a unidirectional offline channel network to buy and sell the contents. In this proposed content propagation method, a user may ask for ‘Proof of path’ from another user who had shared content with it. ‘Proof of path’ ensures that a continuous path (with users who have shared it) exists from the creator of the content to any user who wants to share it.

#### Definition 1

A user *A* can present a ‘proof of path’ to another user *B* by proving that it has paid for a content. A secure and privacy-preserving ‘proof of path’ will hide the identity of the users from whom *A* has bought the content and *A* cannot make a false claim about such purchase, and reuse a ‘proof of path’.

The protocol for secure content propagation in the DSN is as follows:

1. Any node $v_3$ can ask any node $v_2$ for “proof of a path” before it accepts and propagates the content from $v_2$. The “proof of a path” will be proof that $v_2$ has received the content from one of its neighbour $v_1$ in the DSN. However, the proof cannot include information that $v_1$ is neighbour of $v_2$ in the DSN. The protocol for secure content propagation with “proof of path” is shown in Fig. 4:
2. $v_1$ wants to share a content with $v_2$. It sends an overview of the content to $v_2$ and asks $v_2$ if $v_2$ wants to buy the “proof of path” from $v_1$.
3. $v_1$ sends the proof of path $v_2$ and if $v_2$ is satisfied with the proof of path then it will pay $v_1$ for the proof of path.
4. $v_1$ and $v_2$ will choose a common node in the blockchain network controlled by the OSN operator. In this example $v_x$ is such a node. $v_2$ will pay $v_1$ via $v_x$.
5. $v_2$ will inform $v_1$ about the next key to be revealed in the channel from $v_2$ to $v_x$. Let it be the key to the Hash $H^a_2$.
6. $v_1$ will inform $v_x$ that $v_2$ will pay $v_x$ 1/*k* tokens if $v_x$ pays $v_1$ 1/*k* tokens by revealing the key to $H^a_2$.
7. $v_x$ checks if $H'^3_2$ is the next to be revealed by $v_2$ in the channel from $v_2$ to $v_x$. In such a case, $v_x$ agrees to pay $v_1$ 1/*k* tokens and collect 1/*k* tokens from $v_2$.
8. $v_x$ sends a HTLC to $v_1$ which states the following:
  - From the multi-signature address $M_{x,1}$, $1-x*p$ tokens will be given to $v_x$ after time $T-e$ if $v_1$ does not claim these tokens by presenting the key to the Hash $H'^3_x$ and the Hash $H^a_2$. The remaining tokens will be given to $v_x$ if it can reveal the key $v^a_1$. The only difference with the HTLCs exchanged in the unidirectional channel is $v_1$ needs to reveal an additional key $H^a_2$ and the time lock of the HTLC is decreased by *e* (positive number).
9. Next $v_2$ creates a HTLC to $v_x$ as follows:
  - From the multi-signature address $M_{2,x}$, $1-x*q$ tokens will be given to $v_x$ after time *T* if $v_2$ does not claim these tokens by presenting the key to the Hash $H'^2_2$ . The remaining tokens will be given to $v_2$ if it can reveal the key $v^a_x$. It is same as the HTLC exchanged in the unidirectional channel.
10. Next $v_2$ reveal the key to $H^a_2$ to $v_1$ and $v_1$ can use it to collect the tokens from $v_x$ who will reveal the same key to get the token from $v_2$. The key $v^a_2$ will be the “proof of path” that $v_2$ bought from $v_1$. $v_2$ will use to prove to its another neighbour $v_3$ in the DSN that it has received the content from its neighbour in the DSN as follows:
  - $v_2$ will inform $v_3$ about the key to $H^a_2$ and the node $v_x$ controlled by the OSN operator.
  - $v_1$ will ask $v_x$ about uniqueness and validity of $v^a_2$. $v_x$ will inform $v_2$ that $v^a_2$ is a valid “proof of path” if it knows the key to $v^a_2$. However, $v_x$ will send such verification only once to a “proof of path” request for a Hash, i.e. if another neighbour of $v_2$ in the DSN or OSN asks to verify the same “proof of path” then $v_x$ will respond that the “proof of path” is incorrect.
  - After verifying the validity of the “proof” of path $v_3$ may buy this proof of path from $v_2$ via a node in the blockchain network controlled by the OSN operator as shown in steps 2 to 9.

We note the following remarks about the procedure to create DSN in an OSN and the content propagation procedure as follows:

1. The users of the OSN trust the nodes of the blockchain network as the creation of DSN does not require to hide the social neighbourhood of a user in the DSN from the nodes controlled by the OSN operator.
2. The users of the OSN network do not trust other users of the OSN network and the DSN formation procedure must hide the neighbourhood of a user in DSN from all other users.

## Analysis

### Theorem 1

Channels are guaranteed to expire after *k* transactions.

### Proof

As shown in the previous section, uni-directional channels will expire after *k* transfers. The only possibility to improve the longevity of channels is a user transfers funds to another user without involving the OSN operator nodes. But due to the topology of the offline channel network used in this solution, it is not possible (Fig. 4). $\square$

**Fig. 5:**
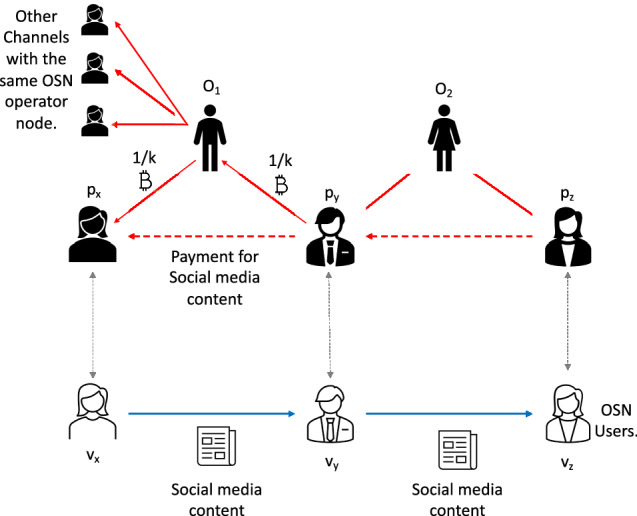
Description of a proof of path

### Theorem 2

The DSN is privacy-preserving.

### Proof

Consider the scenario in Fig. 5. The user $v_y$ presents proof of path to the user $v_z$. The proof of path is the proof that $v_y$ paid $v_x$ for the content. $v_x$ is a social neighbour of $v_y$ in the DSN. In this case, $v_z$ is an attacker who wants to know from whom $v_y$ bought the content or identity of $v_x$. The proof of path will only include the information that $v_y$ paid $O_1$ for the content. $O_1$ can confirm this to $v_z$ and $v_z$ can also check the transaction used to open the channel from $O_1$ to $v_y$. But $O_1$ does not reveal that it paid $v_x$ on behalf of $v_y$. Thus $v_z$ can only guess to whom $O_1$ made the payment on behalf of $v_y$. There will be very few OSN nodes in the blockchain network and each such node will have channels with multiple OSN users. Thus $O_1$ will have multiple channels with other OSN users. Hence $v_z$ cannot identify $v_x$ as a neighbour of $v_y$ in the DSN. $\square$

### Theorem 3

The verification for proof of path is privacy-preserving.

### Proof

In the attack on the privacy of proof of path, the user $v_z$ (Fig. 5) wants to know the identity of $v_x$ in the proof of path presented by $v_y$. $v_z$ can only be successful if it can reveal the social neighbourhood in the DSN. Hence, because of Lemma 2, Lemma 3 holds. $\square$

### Theorem 4

Proof of path is secure.

### Proof

The security problem with proof of path is the user $v_y$ makes a false claim about paying another user $v_x$ (Fig. 5) about paying for the content. Note that proof of path includes the information that $v_y$ paid $O_1$ and it includes Key to a Hash (the Hash is included in the transaction funding the channel from $v_y$ to $O_1$). $v_y$ cannot make a false claim for proof of path because:

- $v_y$ cannot provide a wrong Hash and key to $v_z$ as $v_z$ will check the transaction used in opening the channel from $v_y$ to $O_1$ to check the existence of such a Hash.
- $O_1$ is assumed to be trusted and wishes to prevent rumour propagation. Hence it will not support verification with the wrong Hash and key pair.
- $O_1$ will lose fund if $v_y$ uses the same proof of path to multiple users, i.e. $v_y$ bought the content once and wants to sell it multiple times. Thus $O_1$ will maintain the uniqueness of proof of path.

$$\square$$

### Theorem 5

The channel balance of irrational users will become lower than rational users.

### Proof

Both the rational and the irrational users will seek proof of path from their neighbours before buying a content from them. As the proof of path is secure and privacy-preserving, it will reduce the channel balance of any user (except the path consisting of social bots where the last bot can buy it from the first bot). Let the probability that a user in the OSN is a social bot is 1/*p*, a user is an irrational user is 1/*q* and a user is a rational user 1/*r*. Let,

$$\begin{aligned} 1/p + 1/q + 1/r = 1 \end{aligned}$$

and

$$\begin{aligned} 1/r \ge 1/q \ge 1/p. \end{aligned}$$

The scenario when an irrational user cannot sell a content is the one when it bought the content from a social bot or from an irrational user (assuming the irrational users will buy or sell any content) and it wants to sell it to a rational user. The probability of such an event is:

$$\begin{aligned} (1/p + 1/q)\times 1/r. \end{aligned}$$

The scenario when a rational user cannot sell a content is the one when it bought the content from a rational or from an irrational user (assuming the best case scenario for rumour propagation) and it wants to sell it to a social bot. The probability of such an event is:

$$\begin{aligned} (1/q + 1/r)\times 1/p. \end{aligned}$$

We will prove by contradiction. Assume that:

1 $$\begin{aligned} (1/q + 1/r)\times 1/p&> (1/p + 1/q)\times 1/r\nonumber \\ (1/(qp) + 1/(rp))&> (1/(pr) + 1/(qr))\nonumber \\ 1/(qp)&> 1/(qr)\nonumber \\ 1/(p)&> 1/(r)\nonumber \\ \end{aligned}$$

This is a contradiction as we assumed that there are more rational users in the OSN than social bots. $\square$

### Theorem 6

**Fig. 6:**
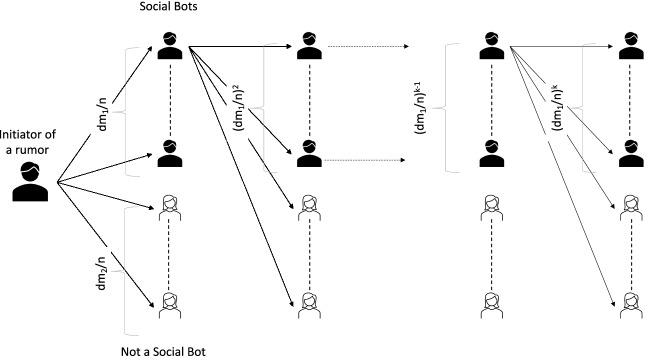
Content propagation with DSN

Content propagation with DSN will prevent the social bots from spreading rumours as fewer users (not social bots) will be influenced and share a rumour (Fig. 6).

### Proof

We calculate the probability that a user (who is not a social bot) will forward the content endorsed by the social bots as follows:

- Let there are *n* users of the social network with $m_1$ social bots and $m_2$ ordinary users ($m_1 + m_2 = n$). Also, let the average degree is *d* and each user needs at least *K* neighbours to endorse a content before it shares.
- Consider the case for the user $v_x$ at a minimum distance 1 from the social bot $v_y$ who had started a rumour.
- $v_y$ has $dm_1/n$ social bots and $dm_2/n$ ordinary users as its neighbours. Each of such social bots have $dm_1/n$ social bots as its neighbours.
- Thus $v_x$ has to choose *d* neighbours from a set of $d+d^2$ users with $dm_1/n +(dm_1/n)^2$ social bots in it. $v_x$ will share the content if among the *d* users it chooses as its neighbours has *k* social bots.
- There are $$\left( {\begin{array}{c}1+d+d^2\\ k\end{array}}\right)$$ combinations to choose *k* users from the set of users $1+d+d^2$. And, there are $$\left( {\begin{array}{c}1+dm_1/n+(dm_1/n)^2\\ k\end{array}}\right)$$ combinations of *k* users from the same set where all chosen users are bots.
- Thus the probability that $v_x$ will be influenced is
  $$\begin{aligned} \left( {\begin{array}{c}1+dm_1/n+(dm_1/n)^2\\ k\end{array}}\right) /\left( {\begin{array}{c}1+d+d^2\\ k\end{array}}\right) . \end{aligned}$$
- Thus the expected number of ordinary users who are at a distance 1 and who will share the content is:
  $$\begin{aligned} (dm_2/n)\times \left( {\begin{array}{c}1+dm_1/n+(dm_1/n)^2\\ k\end{array}}\right) /\left( {\begin{array}{c}1+d+d^2\\ k\end{array}}\right) . \end{aligned}$$
- Using similar calculation we can find the minimum number of expected ordinary users at distance *z* from the social bot who initiated sharing the rumour who will share the content is:
  2 $$\begin{aligned}&(dm_2/n)^z\times \left( {\begin{array}{c}d'^{z-1}+d'^z+d'^{z+1}\\ k\end{array}}\right) / \left( {\begin{array}{c}d^{z-1}+d^z+d^{z+1}\\ k\end{array}}\right) \end{aligned}$$
- In presence of the DSN, the probability that any neighbour chosen at random will be a social bot is $m_3/n<m_1/n$.
- Expected number of ordinary users who will share the content at distance *k* is
  $$\begin{aligned} (dm_2/n)\times \left( {\begin{array}{c}1+dm_3/n+(dm_3/n)^2\\ k\end{array}}\right) /\left( {\begin{array}{c}1+d+d^2\\ k\end{array}}\right) . \end{aligned}$$
  as $m_3<m_1$, the number of ordinary users who will share the content will be less.

$$\square$$

## Evaluation

We will use simulations of content propagation in social chains to evaluate the proposed method to prevent social bots from spreading rumours. We will use agent-based modelling to develop such a simulation. The pseudocodes of these simulations are shown in Algorithm 1 and 2 and as follows:

1. Upon receiving content, a user who is not a social bot will share the content if the number of neighbours who have shared the content is more than a threshold $(\tau )$.
2. Upon receiving content, a user who is a social bot will share the content with all of its neighbours.
3. In presence of the DSN, upon receiving content, a user who is not a social bot will share the content if the number of neighbours in the DSN who has shared the content is more than a threshold $(\tau )$.
4. In presence of the DSN, upon receiving content, a user who is a social bot will share the content with all of its neighbours.

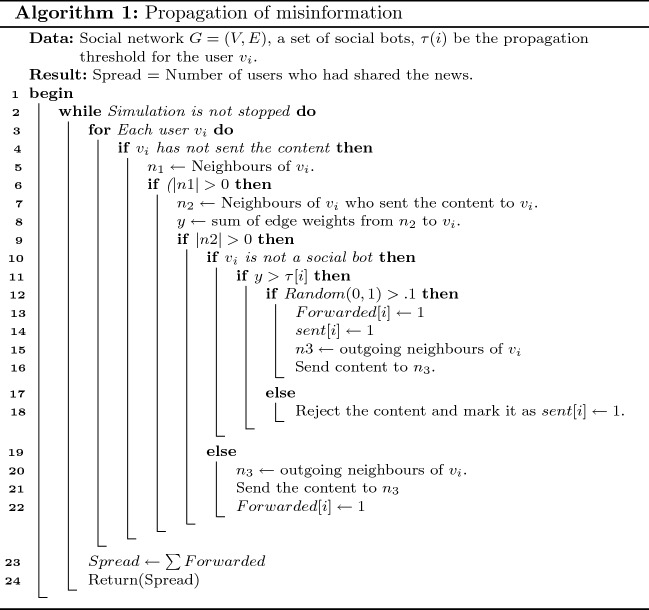

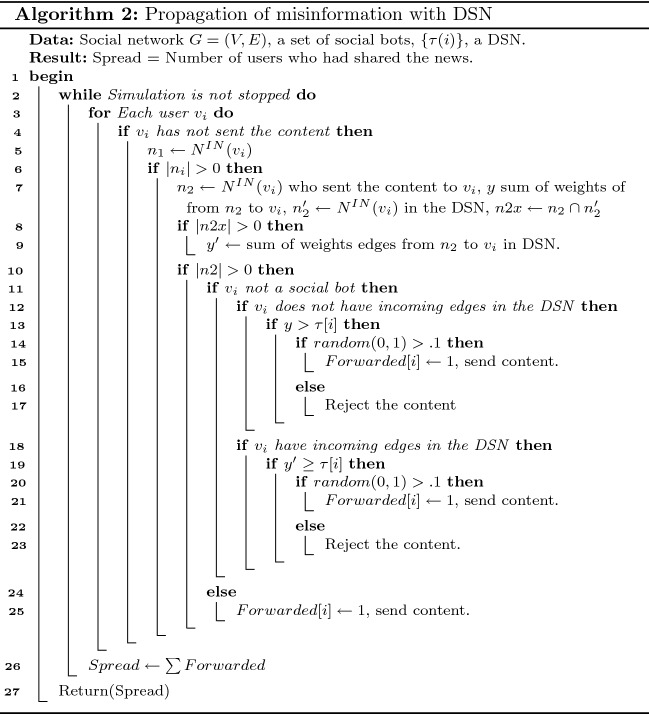

We used the Facebook data set from Leskovec and Mcauley (2012). We created bidirectional edges for every edge in this dataset. The resultant directed graph represents a social network, and it has 4039 nodes and 176468 edges. The graph has an average degree of 87. We will evaluate the performance of the proposed method to prevent social bots from spreading rumours with the following parameters:

1. DSN size: It denotes the fraction of neighbours in the OSN who a user trusts and considers as a social neighbour in the DSN.
2. Threshold ($\tau$): It denotes the weighted number of neighbours who must share the content with a user before the user is influenced and shares the content. All neighbours of each user are given a weight (positive fraction), and the sum of such weights for each user is 1.
3. Number of social bots: We will vary the number of social bots in the social network. We will assume that any user is a social bot with the probability *m*/*n* where *m* is the total number of social bots and *n* is the total number of users.

**Fig. 7:**
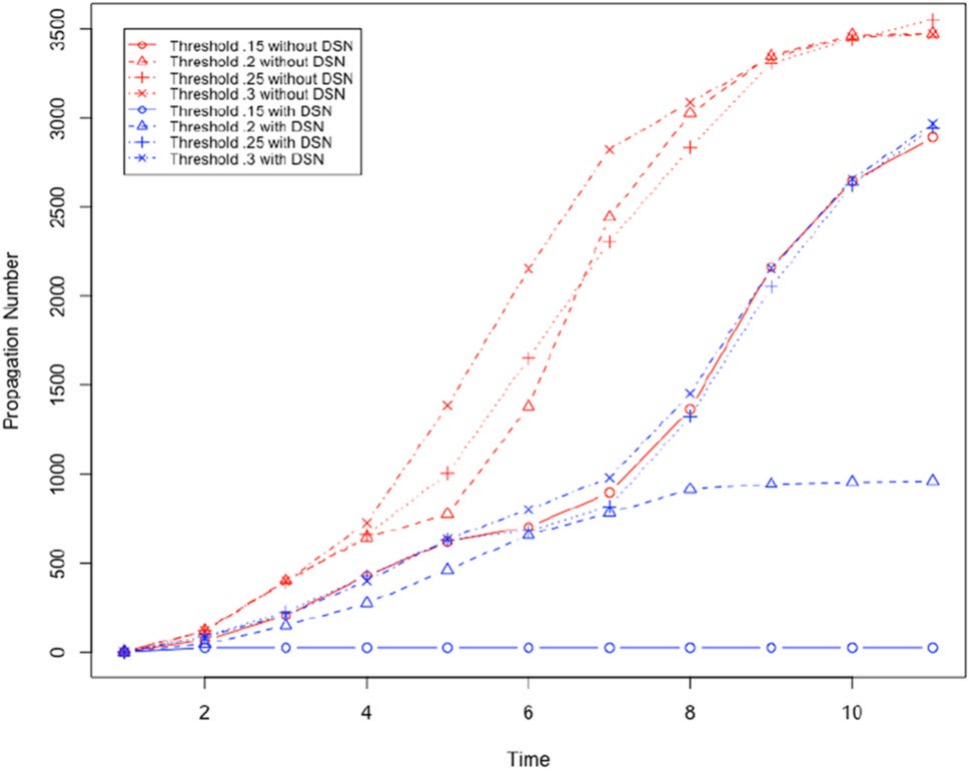
It shows the number of users who shared the content where $\tau$ is gradually increased and rumour start node is 2544

**Fig. 8:**
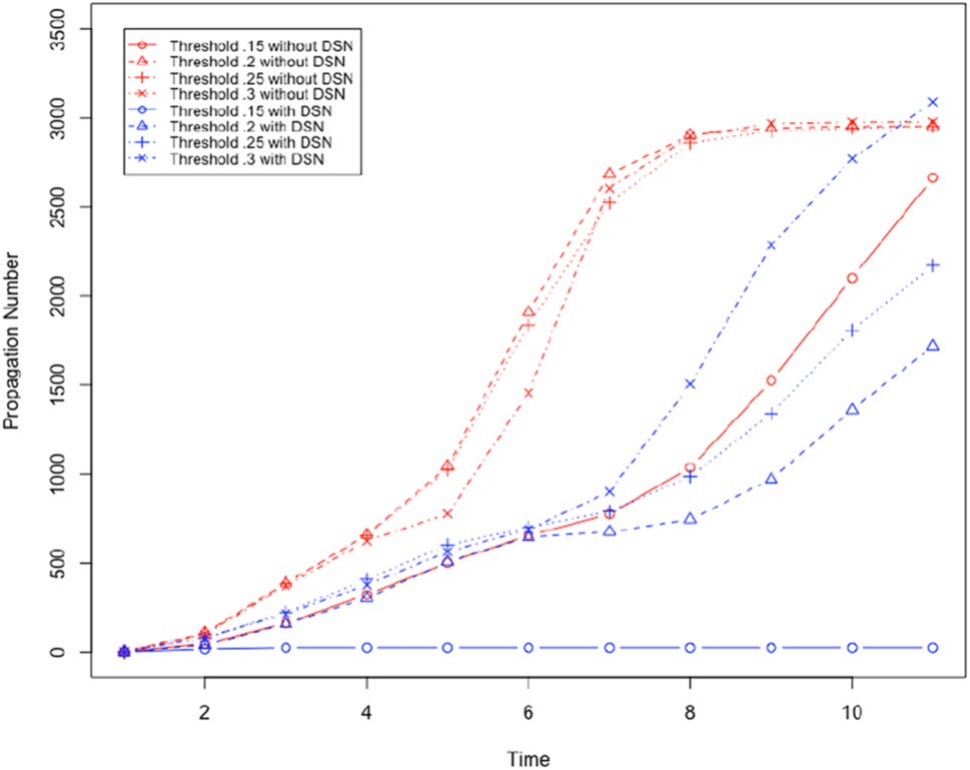
It shows the number of users who shared the content where $\tau$ is gradually increased and rumour start node is 2626

First, we will evaluate the performance of the proposed method to prevent social bots from spreading rumours by gradually increasing $\tau$. By increasing $\tau$ we make it more difficult to propagate content, i.e. more neighbours have to share content to influence a user. We will use two social bots as the initiator of the rumour. The social bots are nodes 2626 and 2544 with a degree 372 and 588. Next, we create a DSN where each user (not social bots) chooses 20% of its neighbours in the OSN as neighbour in the DSN. Each user (not social bots) chooses a social bot as its neighbour in the DSN with probability .1, and it chooses another user who is not a social bot with probability .9. It means each user (not a social bot) carefully chooses its neighbours in the DSN. Using these parameters, we execute a set of 4 executions of the content propagation simulation. In every execution of the simulation, we execute the simulation of content propagation with and without the DSN by choosing $\tau$ .15,.2,.25, and .3. We measure the number of users who share the content as the propagation number. Figures 7 and 8 show the outcome of the simulation execution where the rumour spreading is started by node 2544 and 2626, respectively. In these results we observe the following:

1. For all tested values of $\tau$, the proposed method to prevent rumour spread is successful. Rumour propagation is very low (less than 50) for $\tau \le .15$.
2. Performance of the proposed method to stop rumour propagation works better with decreasing value of $\tau$.

Next, we evaluate the performance of the proposed solution to prevent rumour propagation by changing the size of the DSN. We execute four sets of experiments by increasing DSN size from 15% (i.e. 15% edges from OSN is chosen in the DSN) to 30%, while $\tau$ remains .15. The selected DSN is a disconnected graph with the following parameters:

**Table:** 

DSN size	Number of Clusters in the DSN	Average degree
.15	262	26
.2	220	34
.25	197	43
.3	170	52

**Fig. 9:**
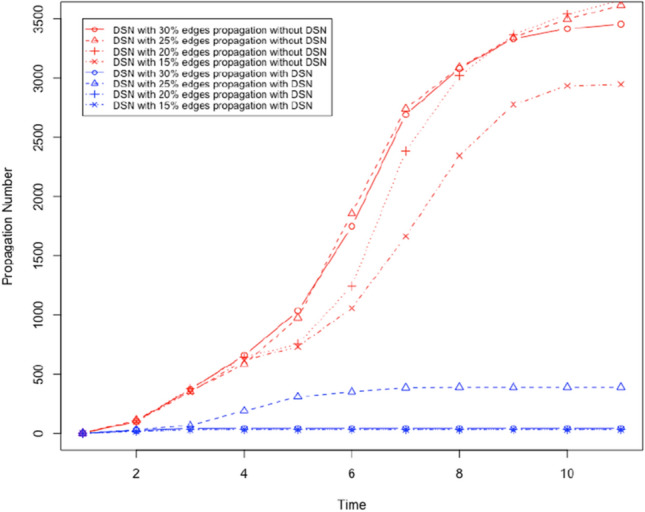
It shows the number of users who shared the content where DSN size is gradually increased and rumour start node is 2544

**Fig. 10:**
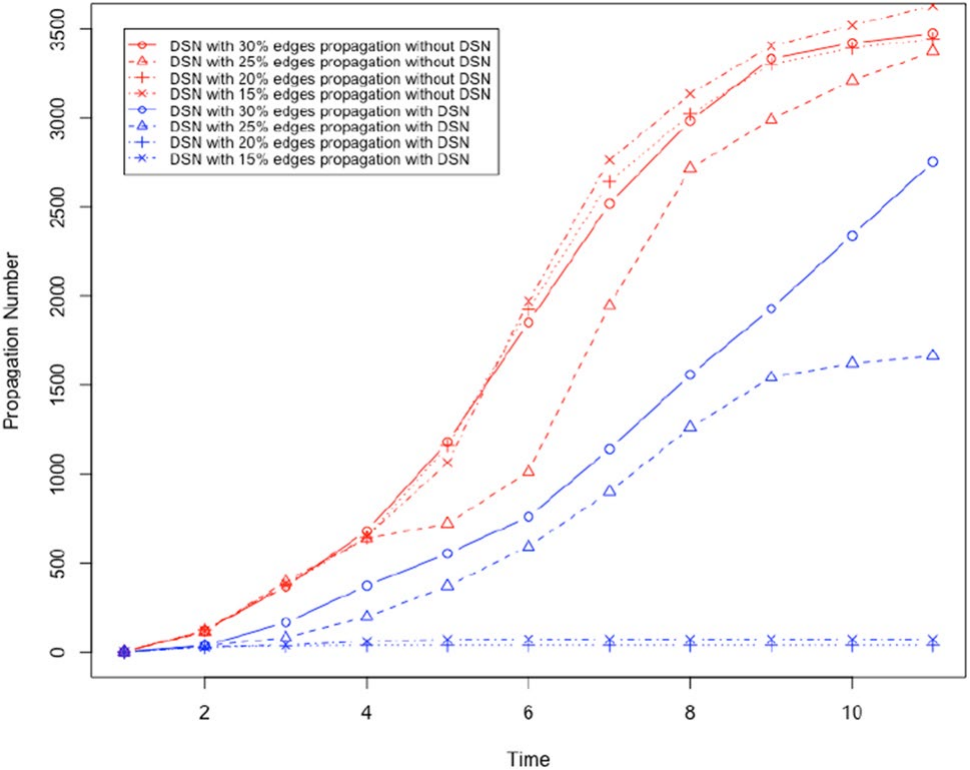
It shows the number of users who shared the content where DSN size is gradually increased and rumour start node is 2626

The results are shown in Figs. 9 and 10 . We observe the following:

1. For all tested values of DSN size (number of edges in the OSN chosen as the DSN), the proposed method to prevent rumour spread is successful as it significantly reduces the number of users who shared the rumour. Rumour propagation is very low (less than 50) for DSN size more than $20\%$.
2. As we increase the DSN size, the performance of the proposed method to stop rumour propagation improves.

**Fig. 11:**
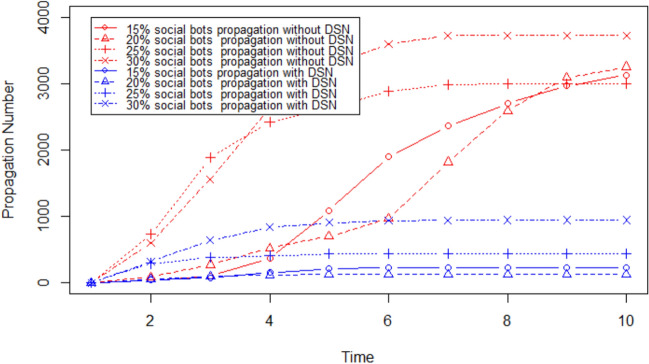
It shows the number of users who shared the content where the number of social bots is gradually increased

Next, we evaluate the performance of the proposed solution to prevent social bots from spreading rumours by increasing the number of social bots. We execute four sets of simulations with $\tau = .2$, and DSN size is 15%. We increase the number of social bots from 15% to 30% in these experiments. The outcome of these experiments is shown in Fig. 11. We observe the following:

1. For all tested numbers of social bots, the proposed method to prevent rumour spread is successful as it significantly reduces the number of users who shared the rumour. Rumour propagation is very low (less than 50) for DSN size more than $20\%$.
2. As we increase the number of social bots, the performance of the proposed method to stop rumour propagation gets worse. However, with a maximum 30% social bots the proposed solution can decrease the number of users who share the rumour by only 75%.

**Fig. 12:**
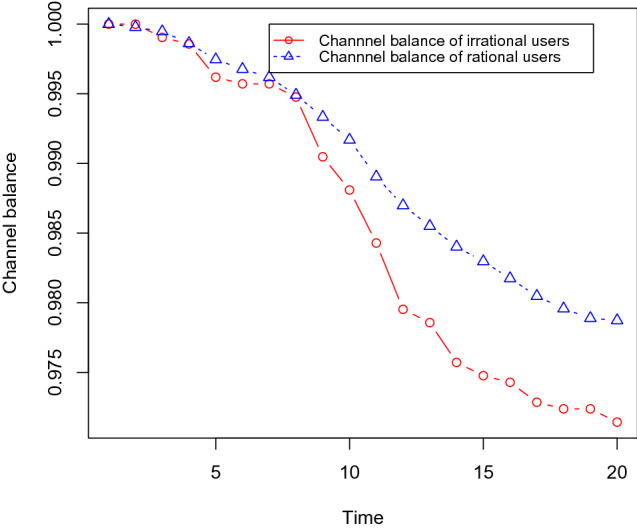
It shows that the channel balance of irrational users becomes lower than the same for the rational users. We randomly choose 10% of the OSN users (who are not social bots) as irrational users

**Fig. 13:**
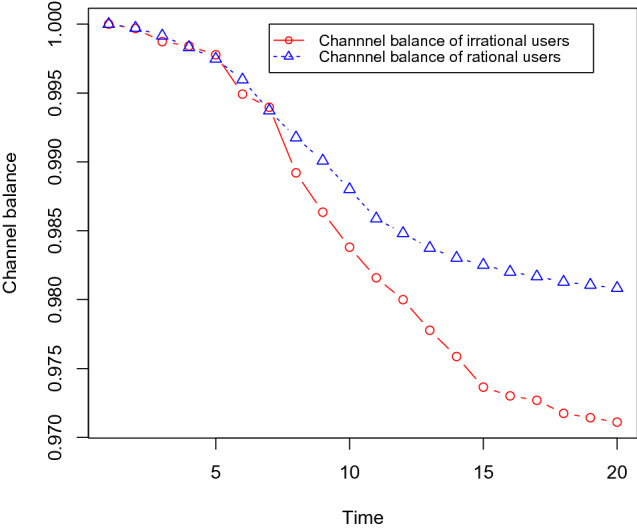
It shows that the channel balance of irrational users becomes lower than the same for the rational users. We randomly choose 15% of the OSN users (who are not social bots) as irrational users

**Fig. 14:**
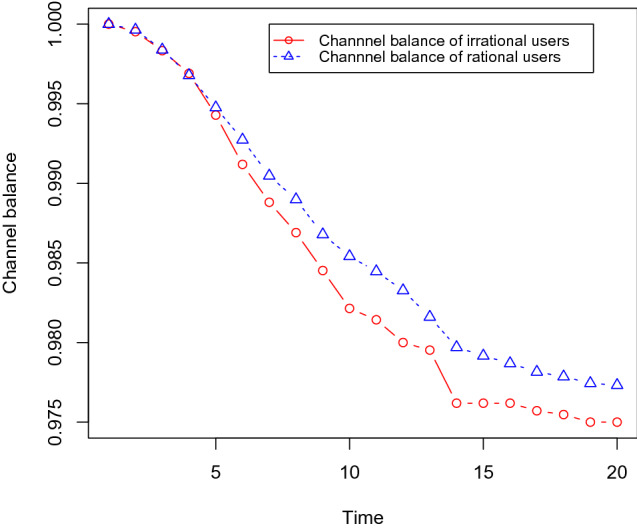
It shows that the channel balance of irrational users becomes lower than the same for the rational users. We randomly choose 20% of the OSN users (who are not social bots) as irrational users

**Fig. 15:**
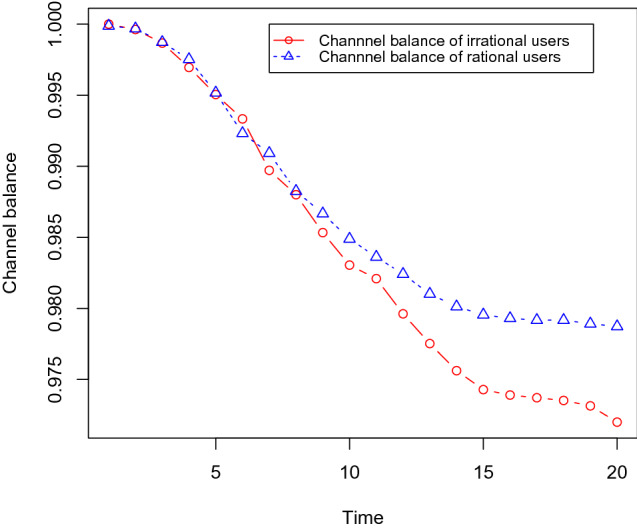
It shows that the channel balance of irrational users becomes lower than the same for the rational users. We randomly choose 25% of the OSN users (who are not social bots) as irrational users

Next, we show that the channel balances of irrational users become lower than the same for rational users. We use the same dataset as before with 30% social bots, and we gradually increase the number of irrational users as 10, 15, 20, and 25%. Each channel is initialised with one token and it takes 0.1 tokens for each transfer in the channels, i.e. it takes 0.1 tokens to buy any content. We measure the channel balance of the users after executing the content propagation simulation with these different sets of irrational users. We found that the channel balance of irrational users becomes low compared with rational users. These results are shown in Figs. 12,13,14, and 15 .

## Conclusion

Social bots can spread misinformation in OSN to create social, political, and economic disruptions. A centralised method to prevent social bots from spreading rumours can be biased and may not be trusted by the users. In this paper, we proposed a decentralised method to prevent social bots from spreading rumours. In this method, the users of the OSN create a DSN using a public blockchain. In this paper, we used Bitcoin as the blockchain platform to form the DSN. We used blockchain offline channels to develop a high scale DSN. We proved that the proposed method is secure and privacy-preserving. We proved that the proposed method can significantly reduce the spread of rumours using simulations of social networks. In the future, we will investigate the cost to execute the proposed method in terms of the blockchain transaction cost.
